# Single-cell atlas of early human brain development highlights heterogeneity of human neuroepithelial cells and early radial glia

**DOI:** 10.1038/s41593-020-00794-1

**Published:** 2021-03-15

**Authors:** Ugomma C. Eze, Aparna Bhaduri, Maximilian Haeussler, Tomasz J. Nowakowski, Arnold R. Kriegstein

**Affiliations:** 1 Department of Neurology, University of California, San Francisco (UCSF), San Francisco, CA USA; 2 The Eli and Edythe Broad Center of Regeneration Medicine and Stem Cell Research, University of California, San Francisco (UCSF), San Francisco, CA USA; 3 Genomics Institute, University of California, Santa Cruz, Santa Cruz, CA USA; 4 Department of Anatomy, University of California, San Francisco (UCSF), San Francisco, CA USA

**Keywords:** Developmental neurogenesis, Cell type diversity

## Abstract

The human cortex comprises diverse cell types that emerge from an initially uniform neuroepithelium that gives rise to radial glia, the neural stem cells of the cortex. To characterize the earliest stages of human brain development, we performed single-cell RNA-sequencing across regions of the developing human brain, including the telencephalon, diencephalon, midbrain, hindbrain and cerebellum. We identify nine progenitor populations physically proximal to the telencephalon, suggesting more heterogeneity than previously described, including a highly prevalent mesenchymal-like population that disappears once neurogenesis begins. Comparison of human and mouse progenitor populations at corresponding stages identifies two progenitor clusters that are enriched in the early stages of human cortical development. We also find that organoid systems display low fidelity to neuroepithelial and early radial glia cell types, but improve as neurogenesis progresses. Overall, we provide a comprehensive molecular and spatial atlas of early stages of human brain and cortical development.

## Abstract (web summary)

Eze et al. use single-cell sequencing and immunohistochemical validation to create an atlas of early human brain development. In the telencephalon, they discover a diversity of progenitor subtypes, including two that are enriched in humans.

## Main

The human brain consists of billions of cells across several functionally interconnected structures that emerge from the neuroectoderm. Many of these structures are substantially expanded or distinct compared to other mammals, particularly the cerebral cortex, the outermost layer of the human brain responsible for perception and cognition. These differences emerge at developmental stages before birth, and thus exploring the cell types in the developing human brain is essential to better characterize how cell types across the brain are generated, how they may be affected during the emergence of neurodevelopmental disorders and how human neural stem cells can be directed to specific cell types for modeling or treatment purposes.

The brain exponentially increases in size after the neural tube closes^1^. Later in development, across brain regions, a series of similar neurogenic and gliogenic processes give rise to the constituent cell types. However, at the molecular level, the sequence of events that leads to the emergence of these progenitor cells early in development is less well understood. The diversity of brain structures is known to emerge as a result of segmentation events that generate the prosencephalon, mesencephalon and rhombencephalon that are then further specified into the anatomical structures (telencephalon, diencephalon and so on) that were dissected in this study^2^ (Supplementary Fig. 1a–c). It has been proposed that there are core gene regulatory programs that enable the specification of these regions and subsequent development of topographically relevant cell types^3^. We sought to explore whether our data could more comprehensively define regional signatures and also identify cell type-specific similarities and differences in these nascent brain structures.

The human cerebral cortex is more than three times expanded compared to our closest nonhuman primate relatives^4^. The cortex emerges from an initially pseudostratified neuroepithelium that gives rise to radial glia, the neural stem cells of the cortex^1^. Radial glia generate neurons, initially through direct neurogenesis and then indirectly through transit-amplifying IPCs^5^. A number of subtypes of radial glia have been identified as the cortex matures, and their primary role in neurogenesis declines late in the second trimester, at which point they generate the glial populations of the cortex^6^. Single-cell RNA-sequencing (scRNA-seq) has added substantially to our knowledge about cellular diversity and signaling networks, particularly during stages of peak neurogenesis. However, the first trimester of cortical development has not been described at this level of molecular detail, and important questions remain about the timing of neurogenesis, the presumed uniformity of the neuroepithelium and the signals that promote the transition to radial glia.

## Results

### Whole brain analysis

To identify cell types and trajectories that lay the foundation for the development of the human brain, we performed scRNA-seq using the droplet-based 10X Genomics Chromium platform. We sequenced cells from ten individuals during the first trimester of human development, spanning Carnegie stages (CS) 12 to 22, corresponding to gestational weeks 6–10. We also included cortical samples from one CS13 and one CS22 individual that were analyzed in a previous study^7^. To identify the chief cell populations across brain regions and to compare them to one another, we sampled all available and identifiable structures, including the telencephalon, diencephalon, midbrain, hindbrain, cerebellum, ganglionic eminences, thalamus, hypothalamus and cortex (Supplementary Figs. 1–3 and Supplementary Table 1). We validated that our sequencing did not contain substantial artifacts or cell debri, and that it represented highly expressed transcripts from bulk RNA-seq experiments^8^ (Supplementary Fig. 1). In total, we collected 289,000 cells passing quality control.

Across brain regions, hierarchical analysis initially identified two main cell classes; progenitors and neurons. To identify the developmental region-specific gene signatures for each brain area, including the hindbrain, midbrain, thalamus, ganglionic eminences and cortex, we performed differential gene expression across areas at each age (Supplementary Tables 2–4). Using samples at CS22, we generated data-driven regional signatures and explored when the most structure-specific genes emerged. We observed that characteristic transcription factor expression (such as *HOX* genes (hindbrain)^9^; *PAX7* (midbrain)^10^; *GBX2* (thalamus)^11^; *NKX2-1* (medial ganglionic eminence)^12^ and *FOXG1* (cerebral cortex)^13^) segregated these regions from one another as early as CS13. However, the cell type-defining gene expression programs were largely conserved across brain regions, resulting in subsets of progenitors or neurons that transcriptomically appear similar across regions (Supplementary Fig. 2b). This was reflected by the fact that coclustering at the early stages could not segregate regional identities, but that this became possible at later stages. At the earliest timepoint, CS12, the differences between brain regions were minimal and did not resemble the more advanced region-specific programs that were identifiable later in the first trimester (Supplementary Figs. 2 and 3).

### Single-cell sequencing of the telencephalon

**Fig. 1:**
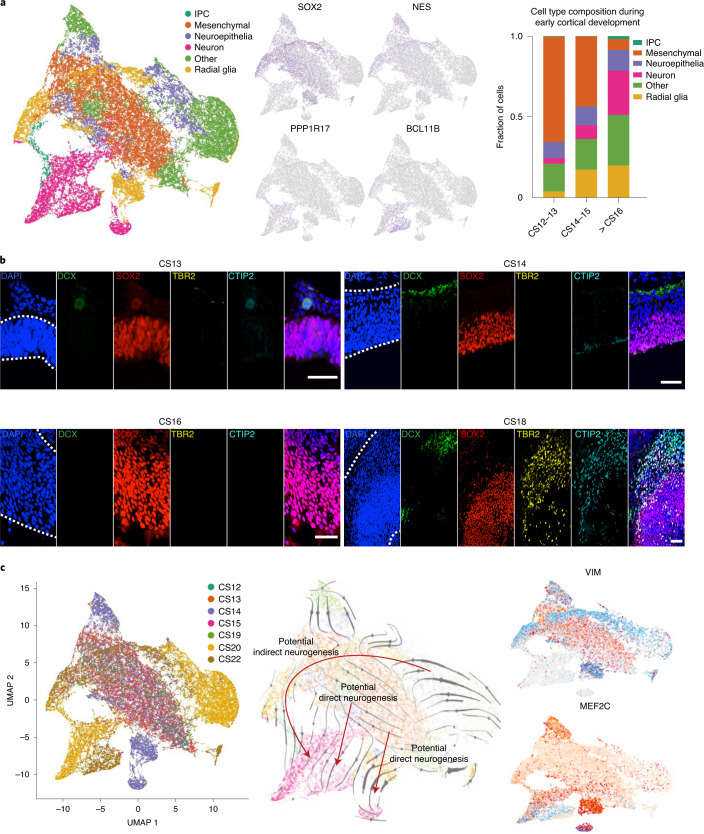
Cell types in the early human cortex. **a**, scRNA-seq of early cortical development. UMAP plot of 58,145 telencephalon or cortical dissections colored by annotated cell type. Feature plots of markers of broad progenitors (*SOX2*), radial glia (*NES*), IPCs (*PPP1R17*) and neurons (*BCL11B*) are shown. Stacked bar chart shows cell type composition at earliest (CS12–13), middle (CS14–16) and late (CS19–22) first trimester. **b**, Spatial immunostaining of early cortical samples. Immunostaining for main cell type markers across first trimester stages. Because of limited sample availability, each sample was immunostained once. Nuclei shown in blue (4,6-diamidino-2-phenylindole (DAPI); dotted line demarcates cortical span), newborn neurons marked by DCX (green), progenitors by SOX2 (red), IPCs by TBR2 (yellow) and maturing neurons by CTIP2 (cyan). Scale bars, 50 μM; in CS13, 25 μM. **c**, RNA velocity demonstrates streams of direct and indirect neurogenesis. UMAP colored by age shows the segregation of samples by early and late first trimester stages. RNA-velocity trajectories are depicted by gray arrows in the middle UMAP plot with underlying color by cell types as annotated in **a**. Line thickness of the arrows indicates the differences in gene signature between cell types, and red arrows show predicted direct and indirect neurogenesis trajectories. The velocity plots on the right show the intensity of scored velocity for a progenitor gene, *VIM*, and a neuronal gene, *MEF2C*. Velocities highlight the distinction between progenitors and neurons in the clustering and velocity analyses.

To better understand the early stages of cortical development, we focused on the 59,000 cells in our dataset that originated from the telencephalon (earliest dissections) and the cortex specifically (when it was identifiable for subdissection). Clustering this data revealed minimal batch effects as most clusters contained contributions from multiple individuals, and clustering segregated the samples based on early and late first trimester stages (Fig. 1 and Supplementary Fig. 4). Each of the 63 identified clusters could be assigned to a cell type identity of neuroepithelial cells, radial glia, intermediate progenitor cells (IPCs), neurons or mesenchymal-like cells. To differentiate between the radial glia and neuroepithelial cells in our transcriptomic data, we examined whether *SOX2*-positive progenitor clusters contained evidence of neurogenic genes, considered to be a criteria of radial glia identity^14^. *SOX2*-positive progenitors that did not have these characteristics were labeled neuroepithelial cells. Additionally, we noted ‘other’ cell populations of endothelial cells, microglia and pericytes (Supplementary Tables 5,6). Furthermore, we observed that the mesenchymal cell population diminished over the course of the first trimester. Comparison of these clusters to previously published single-cell data, including a small number of first trimester cells, showed a strong cluster-level correlation, indicating that our larger dataset presented here recapitulated populations that were previously observed. Additionally, in our previous analysis, some clusters from these early samples were marked ‘unknown’ in identity^15^; our correlation analysis is sufficient to now assign cell type identities to these clusters (Supplementary Fig. 5).

We observed that although neurogenesis ramps up by the end of the second trimester, even the earliest samples contained a small number of neurons (Fig. 1a). It seemed unlikely that they could be migratory populations from other cortical regions since migratory interneurons have not been identified until later developmental timepoints^16^. Presumably these neurons were produced locally by direct neurogenesis from radial glia that occurs early in nonhuman models of cortical development as well as human cortical organoids^17^, and would be consistent with the observation that the IPCs that mediate indirect neurogenesis are nearly absent until later in the first trimester (after CS16). However, without lineage tracing, the inference that the earliest neuronal populations are a product of direct neurogenesis remains a working hypothesis. Subclustering of the neuronal populations resulted in subtype clusters, some of which were strongly enriched in either younger samples (<CS16, presumed to result from direct neurogenesis) or older samples (>CS16) (Supplementary Fig. 6 and Supplementary Table 7). These included expected differences such as clusters marked by *NHLH1*, which was significantly higher in the older samples and has been associated with newborn neurons derived from IPCs^18^. Other genes significantly enriched in older samples included *NEUROD6* and *BCL11B* that are associated with neuronal and deep layer identity^14^, as well as *CALB2* that is expressed in migratory ventrally derived interneurons and a subset of excitatory neurons^19^. We noted significantly higher expression of *MEF2C* in the younger samples. This has been identified as a regulator of early neuronal differentiation and layer formation^20^, but is also a factor involved in synaptic maturation at later stages^21^. We validated high expression of *MEF2C* at the earliest timepoints using in situ hybridization, although some of the expression was extra-cortical. By CS22 its expression had diminished, but *MEF2C* was highly expressed again by mid-gestation at gestational week 14 (Supplementary Fig. 7). This expression pattern is intriguing given the role of MEF2 transcription factors in regulating apoptosis^22^.

We also observed clusters marked by previously undescribed genes, including the younger sample-specific *LHX5-AS1* cluster. We validated that *LHX5-AS1* was strongly enriched at these early timepoints with broad expression at CS13, but restriction to the developing cortical plate by CS14 and CS16 (Supplementary Fig. 8). *LHX5-AS1* RNA had the same expression pattern as LHX5 protein (Supplementary Fig. 9), indicating that it may play a repressive role to the protein, which has been characterized in Cajal–Retzius cell development^23^ but has largely remained unstudied in these early cell populations. The remaining clusters consisted of expected early-born neuronal populations including Cajal–Retzius cells marked by *RELN* (Supplementary Fig. 10), and subplate cells marked by *TLE4* and *NR4A2*. The subplate clusters were unexpectedly heterogeneous (Supplementary Fig. 6c,d) and contained marker genes not previously associated with subplate identity (Supplementary Table 7), suggesting our analysis may provide additional characterization of early neuronal cell type gene expression patterns.

We also sought to describe the spatial organization of the cortical populations across development. Thus, we performed immunostaining validation in five individuals from the first trimester compared to an early second trimester sample at gestational week 14. We observed FOXG1 staining as early as CS16, but not earlier, as has been described previously^24^ (Supplementary Fig. 11). FOXG1 staining ensured that we were identifying cell types in the developing forebrain. We further defined cortical regional and progenitor identity by positive PAX6 expression and positive SOX2 expression. PAX6 expression has previously been identified as a determinant for neuroectoderm fate^25^ and SOX2 as a marker for stem cells. We were also confident of cortical identity based on the anatomical presence of the optic cups (Supplementary Fig. 12) just ventral, lateral and caudal, as well as the nasal ridge, (Supplementary Fig. 12) located just ventral to our regions of interest. Tilescan images of these markers are available for download and exploration in the image browser we created in conjunction with this study (https://cells-test.gi.ucsc.edu/?ds=early-brain, images tab).

In concordance with our transcriptomic data, we observed prevalent SOX2 staining as early as CS13 that persisted through gestational week 14. Newborn neurons marked by doublecortin (DCX) were identifiable as early as CS14, but markers of maturing neuronal identity such as BCL11B (CTIP2) emerged after CS16. As expected, IPCs identified by TBR2 staining did not emerge until CS18 in the dorsal telencephalon^26^ (Fig. 1b and Supplementary Fig. 13), although we and others have observed TBR2 staining at earlier timepoints (CS16) in the ventral telencephalon^27^.

### RNA-velocity analysis of lineage

To characterize the lineage relationship between chief cortical cell types, we performed RNA-velocity analysis using the scVelo algorithm^28^. This algorithm incorporates messenger RNA levels and inherent expression variability of individual cells to infer steady states that contribute to potential lineage relationships. Across cortical cell types, the most apparent lineage stream originated in progenitor cell populations from older samples and followed a trajectory through IPCs to neurons (Fig. 1c). This trajectory of neurogenesis is well described and suggests that the stereotypical process of neuronal differentiation emerges after CS19. At earlier timepoints, there were local examples of radial glia giving rise to neurons, suggestive of potential direct neurogenesis and also lineage relationships between the progenitors themselves. In addition to velocity streams related to the excitatory lineage, there was also a stream from a collection of putative blood–brain barrier cells toward the middle mesenchymal mass. Given the age of these samples, this stream is suggestive of the onset of vasculogenesis that occurs at this time^29^. This is supported by further velocity analysis demonstrating an increase in velocity (indicated in red) of presumptive endothelial (*FN1*) and pericyte (*RGS5*) markers, but not markers of microglia identity (*AIF1*) or the marker of the mesenchymal population (*LUM*) (Supplementary Fig. 4d).

**Fig. 2:**
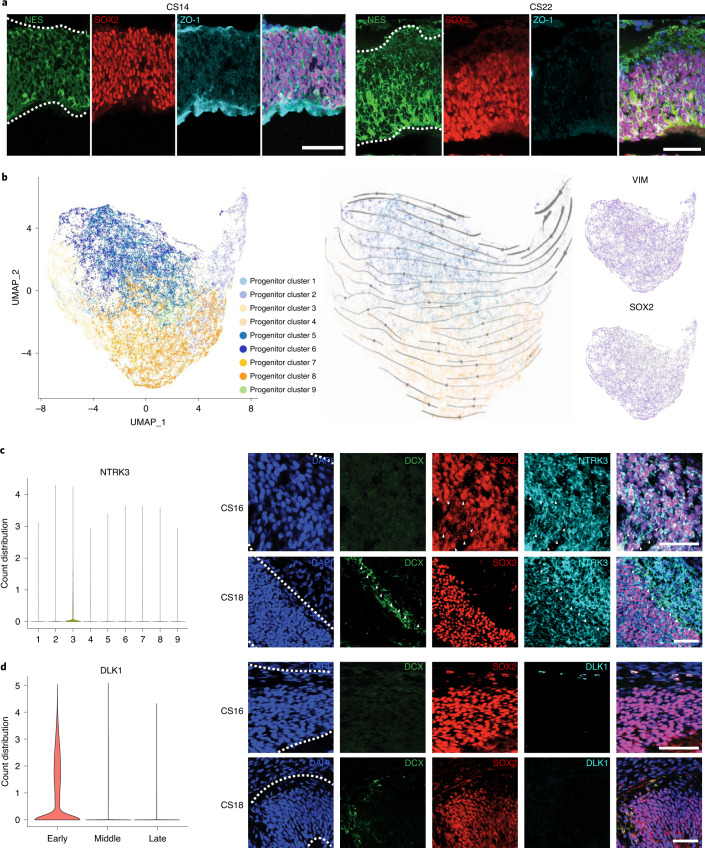
Early progenitors can be divided into nine progenitor subtypes. **a**, Transition from primarily neuroepithelial to radial glia progenitor identity. Immunostaining of early first trimester samples (including CS14, shown here) show strong staining for all progenitors (SOX2, red), as well as tight junctions (ZO-1, cyan), but limited staining for radial glia (NES, green). By CS22, NES staining increases substantially, ZO-1 decreases and SOX2 expression is maintained. Scale bars, 50 μM. **b**, scRNA-seq identifies nine progenitor subpopulations. Left: a UMAP plot depicts subclustered progenitor cells; middle: the velocity trajectory across progenitor subtypes. Right: feature plots show high expression of *VIM* and *SOX2* marking all progenitor populations. **c**, NTRK3 marks progenitor populations before becoming a neuronally enriched marker. Progenitor cluster 3 is specifically and uniquely labeled by *NTRK3*, as shown in the violin plot. Immunostaining for NTRK3 (cyan) shows early labeling of SOX2 (red) positive progenitors at CS16 (white arrows), but expression shifts to more closely coincide with newborn neurons marked by DCX (green) by CS18 (white arrows). Scale bars, 50 μM. **d**, DLK1 marks a subset of early progenitors. DLK1 (cyan) is the top marker for progenitor cluster 8 and is exclusively expressed in early first trimester, as shown in the violin plot on the left. Immunostaining for DLK1 at CS16 shows colocalization with low SOX2 (red) expressing cells at the boundary of the cortical edge. This staining disappears from the cortex entirely by CS18 when DCX (green) staining emerges. For all panels, each sample was immunostained once.

The transition from neuroepithelial cells to radial glia has traditionally been characterized by the reorganization of tight junctions and the appearance of nestin RC2 (NES) immunoreactivity^30^. We sought to visualize this process and its transition across our samples. Using NES as a marker of radial glia, TJP1 (ZO-1) as an indicator of neuroepithelial cells, and SOX2 as a label for all progenitor populations, we saw small numbers of nestin-positive radial glia at CS14, with substantial upregulation by CS22 that simultaneously corresponded to a waning of ZO-1 (Fig. 2a). By gestational week 14, ZO-1 staining dissipated and was largely constrained to presumed vascular structures. During the first trimester, we found a progressive, and incremental, shift away from neuroepithelial identity (Supplementary Fig. 14). To better understand the heterogeneity and spatiotemporal trajectories of neuroepithelial and radial glia progenitor populations, we subclustered the progenitors. Because the mesenchymal population also expressed strong progenitor cell markers including *VIM* and *SOX2*, we removed all neurons, IPCs and other cell populations (including microglia, pericytes and endothelial cells) and subclustered the remaining cells. The resulting analysis yielded nine subclusters all marked by strong *VIM* and *SOX2* expression (Fig. 2b). Each of the nine clusters contained some cells from all first trimester age samples, suggesting that the velocity and maturation trajectories were not purely a function of age (Supplementary Fig. 15b and Supplementary Table 8). To examine if these trajectories correspond to expected cell identity transformations from neuroepithelial cells to radial glia, we explored the expression of *HES5* and *FGF10*, which have been described to mediate the transition between these progenitor populations^31,32^. As expected, *HES5* was strongly enriched in radial glia compared to other cell types, and *FGF10* peaked in neuroepithelial populations (Supplementary Fig. 15). We explored whether any of these progenitors expressed gene signatures that defined excitatory neurons across cortical areas, and found that three-quarters of the progenitors uniquely expressed an areal identity while the rest were unspecified (Supplementary Fig. 15). We additionally performed weighted gene coexpression network analysis (WGCNA) of the progenitor clusters as an orthogonal metric to identify distinct populations^15,33^. We found strong correspondence between clusters derived from WGCNA analysis and our nine progenitor subtypes, and also found strong enrichment for radial glia-like networks in later stage samples (Supplementary Fig. 15 and Supplementary Table 9).

To examine whether this progenitor distribution was distinct at the earliest timepoints of our dataset, we subclustered the CS12 and CS13 samples individually. From this analysis we identified 29 populations that were characterized by *SOX2* (progenitor), *TOP2A* (dividing), *LHX5-AS1* (previously uncharacterized cell population) and *LUM* (mesenchymal cells). Comparison of these subpopulations to the nine progenitor subtypes identified across all samples demonstrated strong correspondence to these nine cell types, with additional heterogeneity in the CS12 and CS13 mesenchymal populations (Supplementary Fig. 5b and Supplementary Table 10). Velocity analysis identified a strong gradient across the populations from cluster 2 toward clusters 1 and 6. These data strongly indicated a gradient of identity across the nine progenitor populations (Supplementary Fig. 15d). We characterized the genes across all our velocity analyses that most strongly influenced the velocity stream (and thus would be hypothesized to be the chief regulators of cell fate transition). The drivers of velocity were enriched for genes that have been associated with neurodevelopmental or psychiatric disorders, including autism, cortical malformations and schizophrenia (Supplementary Table 11), indicating that very early events in cortical development may result in susceptibility or vulnerability to these disorders. More work at these early timepoints is required to fully characterize the developmental implications.

### Progenitor heterogeneity

To explore the timing and spatial localization of each of the progenitor subpopulations, we performed immunostaining of the most specific gene markers for each cluster (Supplementary Figs. 7–10 and Supplementary Figs. 16–26). Each of these immunostainings is available as a tilescan of the entire brain section in our image browser (https://cells-test.gi.ucsc.edu/?ds=early-brain, images tab). *NTRK3* was a highly specific marker of Progenitor Cluster 3, and intrigued us because it is commonly described as a TrkC receptor that enables survival of specific neuronal populations^34^ and has broad neocortical expression at later stages of mouse cortex development^35^. We observed that at early stages (CS14, CS16), *NTRK3* broadly colocalized with SOX2-positive progenitor cell populations (Fig. 2c and Supplementary Fig. 16), but after CS18, it shifted from progenitor to neuronal expression as would be expected. *DLK1* was a top marker gene for Progenitor Cluster 8 and also was highly enriched in early samples. Although much of the DLK1 staining was in stromal cells peripheral to the cortex, as has been previously reported^36^, we observed a small number of *DLK1*-positive cells that expressed low SOX2-levels at early stages, such as CS16, after which *DLK1* expression completely disappeared (Fig. 2d and Supplementary Fig. 17). *DLK1* is a noncanonical ligand of Notch that inhibits its function and affects Notch 1 receptor distribution^37^, although it is not widely expressed in the mammalian central nervous system during development^38^. *DLK1* has also been described to play a role in enabling postnatal neurogenesis in the mouse^39^, and may play a similar regulatory role at the earliest neurogenic timepoints of human cortical development. Both *NTRK3* and *DLK1* may play important roles in receptor/ligand communication, as integration with other brain regions increases (Supplementary Table 12). Across progenitor populations we describe known and new gene expression patterns that define the neuroepithelial and radial glia subpopulations and suggest that there exists an expression gradient that marks the transition from early to more mature progenitor populations.

**Fig. 3:**
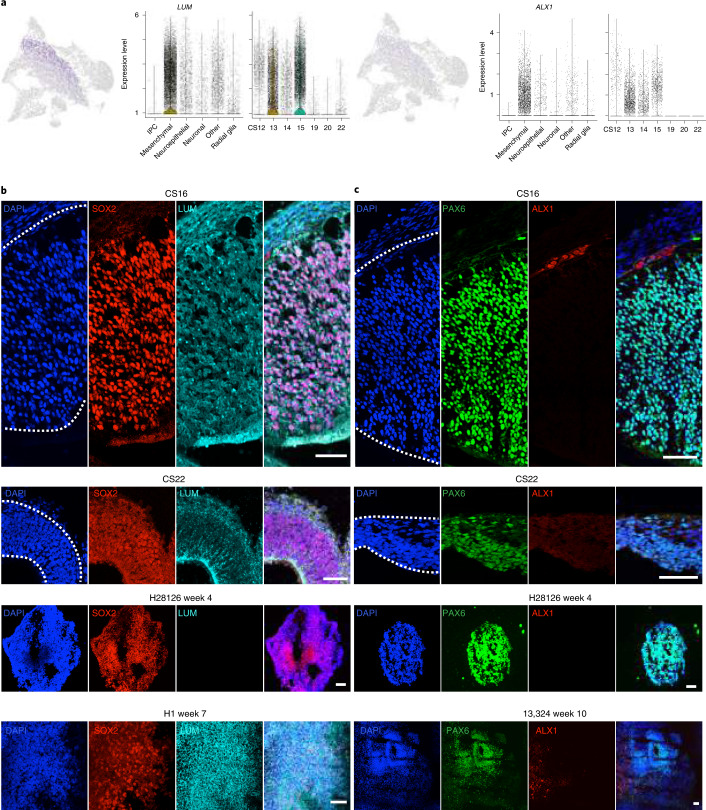
scRNA-seq identifies early mesenchymal cell population. **a**, *LUM* and *ALX1* are mesenchymal cell type and early sample markers. In both progenitor and cortex clusterings, *LUM* marks a separate population of cells, as shown by the feature plot on the left. *LUM* expression is highly specific to the mesenchymal cell type and is enriched in early samples. *ALX1* expression is highly correlated to *LUM* as shown in the right feature plot, and is similarly enriched in early, mesenchymal populations. **b**, *LUM* is widely expressed early and diminishes later. Immunostaining for *LUM* (cyan) shows prevalent expression in and between progenitors marked by SOX2 (red) at CS16, but this expression dissipates by CS22. However, expression of *LUM* does not begin until week 7 in the H1 organoid. Scale bars are 50 μM. **c**, *ALX1* is sparsely expressed early and diminishes later. Immunostaining for *ALX1* (cyan) shows sparse expression in PAX6 (green) positive cells; it disappears from the cortex but is expressed in surrounding brain structures at CS22. *ALX1* expression does not begin until week 10 in the 13,234 cerebral organoids. For all panels, each sample was immunostained once. Scale bars, 50 μM.

Both in our initial clustering and in the more focused progenitor analysis, a mesenchymal-like population labeled by the gene *LUM* was distinctly segregated from other progenitor populations. Lumican, the protein encoded by *LUM*, is an extracellular matrix protein that is widely present in mesenchymal tissues throughout the adult body^40^. It has also been used to promote folding in cortical structures when added exogenously to media^41^. In our data, *LUM* expression is highly enriched in the mesenchymal cell population and diminishes substantially after early developmental timepoints (Fig. 3a). Another marker of this cluster was *ALX1*, which also has one of the most correlated gene expression patterns to *LUM* (Fig. 3b). *ALX1* has been described as required in knockout mice for the development of the forebrain mesenchyme^42^, but has not been studied in humans. We observed prevalent *LUM* staining in our samples at or before CS16, with *ALX1* at the edges of the forebrain, including several colocalized PAX6-positive cells (Fig. 3c,d and Supplementary Figs. 18 and 19). *ALX1* staining substantially diminishes later in development.

### Canonical developmental signaling pathway activation

**Fig. 4:**
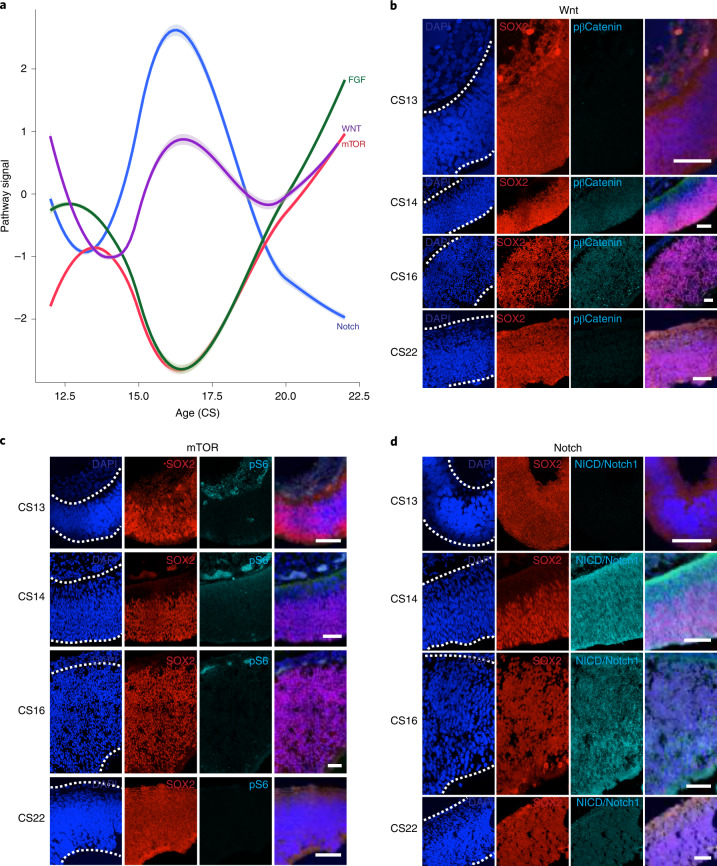
Signaling pathway oscillations in the first trimester human cortical progenitors. **a**, Signaling pathway oscillations in progenitor RNA expression patterns of the FGF (green), Wnt (purple), mTOR (red) and Notch (blue) signaling pathways, as defined by KEGG pathway designations in progenitors across ages sampled in this study. Error shading surrounding each bar indicates the loess regression 5–95% confidence interval. **b**, Wnt activity, as indicated by phosphorylated β-catenin (cyan), is highest in the CS14 and CS16 samples and colocalizes with progenitors (SOX2, red), but dissipates by CS22. **c**, mTOR activity, as indicated by phosphorylated S6 ribosomal protein (cyan), primarily localizes along the cortical plate (DCX, green) in the youngest samples and diminishes by CS22. **d**, Notch activity, as indicated by cleaved Notch intracellular domain (NICD) of Notch 1, peaks in expression at CS14 and localizes mainly to the ventricular zone. Nuclei are labeled by DAPI (blue). For all panels, each sample was immunostained once. Scale bars, 50 μm.

Because canonical signaling pathways have been characterized for their role in patterning the human brain and cortex, we sought to explore the dynamics of their expression patterns in human cortical progenitors. scRNA-seq data showed dynamic expression of the genes that have been described as part of the FGF, Wnt, mTOR and Notch signaling pathways (Fig. 4a) in cortical progenitors. The FGF signaling family has been described to assign rostral identity in the developing neural tube. But FGF also plays a role in the transition to and maintenance of the radial glia progenitor pool, in part promoted by FGF10, by permitting the transition of neuroepithelial cells to radial glia and by preventing the transition into intermediate progenitors^32^. Furthermore, FGF signaling interacts with the Notch signaling pathway during cortical development. Classic studies have demonstrated that Notch signaling is generally required to preserve stem cell pools. Constitutive Notch 1 activation increases radial glial generation and leads to a decrease in the expression of proneural genes, such as Neurogenin-2 (Ngn2)^43^. Consistent with this observation, here we find that Notch 1 staining peaks and is largely restricted to the ventricular zone at CS14 and CS16 (Fig. 4d).

mTOR activity in the developing forebrain has largely been uncharacterized at early developmental timepoints, and descriptions of its function have been restricted to its role in regulating oRG cells^15^. However, upregulation of CDC42-dependent mTOR signaling has been shown to be sufficient to generate neural progenitors, mediated through increased HES5 and PAX6 expression^44^, suggesting that mTOR signaling early on may be important for driving the switch from neuroepithelial stem cell to radial glia. We find that expression of phosphorylated S6 is highest in the earliest samples and then diminishes later in the first trimester (Fig. 4c), further supporting a role in the neuroepithelial to radial glia fate transition. Furthermore, Wnt signaling has also been implicated in medial-lateral patterning of the neural tube and in cell fate transitions. Constitutive Wnt activation leads to opposing actions of increasing the progenitor pool by preventing neurogenesis but also of increasing the neuronal pool by promoting IPC differentiation^45^. This suggests that Wnt activity is variable and cell-dependent in cortical development. In this study, we discover that active Wnt signaling (phosphorylated β-catenin) peaks at CS16 and diminishes subsequently (Fig. 4b). These data further clarify our understanding of signaling in patterning cortical progenitors, with important implications for better modeling early stages of cortical development.

### Conservation of progenitor populations across species

**Fig. 5:**
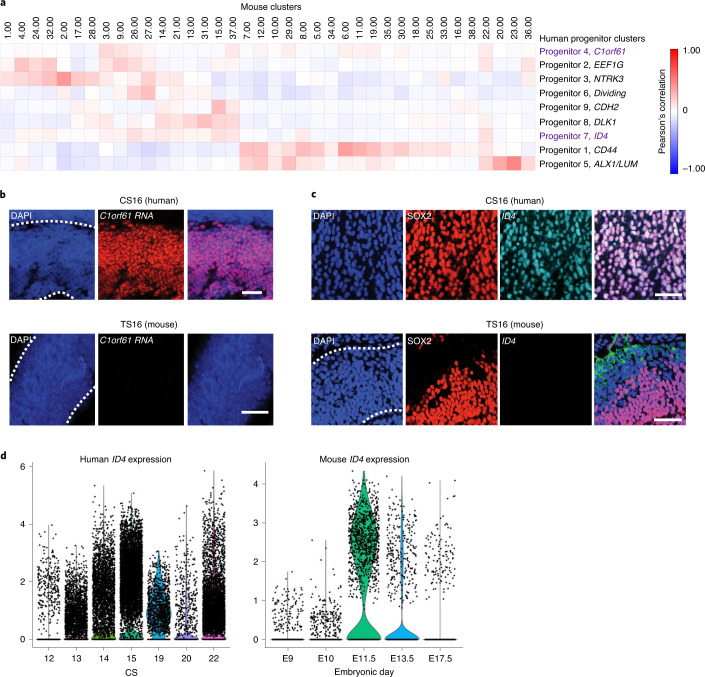
Mouse models different aspects of early cell types. **a**, Comparison of mouse forebrain clusters to primary progenitor cell types. Heatmap showing the comparison of mouse forebrain clusters from 16,053 cells to the primary progenitor populations identified in this dataset. Correlations are performed using Pearson correlations between cluster marker sets and identify that progenitor clusters 4 and 7 do not have a counterpart in mouse data. **b**, *C1orf61* is widely expressed in early human but not early mouse progenitors. Fluorescent in situ hybridization of human samples at CS16 shows broad expression of *C1orf61* (cyan) in progenitor cells labeled by SOX2 (red). Parallel in situ staining in TS16 mouse shows no *C1orf61* expression. Scale bars, 50 μM. **c**, *ID4* is widely expressed in early human but not early mouse progenitors. Immunostaining of human samples at CS16 shows broad expression of *ID4* (cyan) in progenitor cells labeled by SOX2 (red). A parallel staining in TS16 mouse shows no *ID4* expression. Scale bars, 50 μM. **d**, Violin plots of *ID4* RNA expression across several CSs (human) and embryonic days (mouse). *ID4* RNA expression in the human persists onward from CS13. However, *ID4* RNA expression in the mouse peaks at E11.5 (TS20) and dissipates. For all panels, each sample was immunostained once.

To evaluate the similarities and differences between early forebrain development in human and rodent, we performed scRNA-seq of the mouse forebrain at embryonic days (E) 9 and 10, which correspond to Theiler stages (TS) 14 and 16. Although the onset of neurogenesis differs between human and mouse^46^, we used immunostaining to verify that at these mouse stages, the forebrain was already expressing FOXG1 and undergoing neurogenesis, as marked by the presence of DCX (Supplementary Figs. 11 and 13). To compare mouse and human progenitor populations, we performed clustering and correlation analysis between the mouse clusters and our nine human progenitor populations. Using a correlative threshold, we observed that seven of the nine human populations had at least one corresponding cluster in the mouse single-cell data (Fig. 5a). However, there were no high correlations for Progenitor Cluster 4 (marked most highly by *C1orf61*) or Progenitor Cluster 7 (marked most highly by *ID4*). To test the hypothesis that these populations were underrepresented in the mouse data, we used immunostaining to explore the expression of the main progenitor population markers in the mouse. Indeed, no fluorescent in situ signal for *C1orf61* or immunostaining signal for Id4 was identifiable in the mouse forebrain at TS14 or 16 (Fig. 5b and Supplementary Figs. 20–22). Although the TS14 and 16 samples were more advanced in terms of neurogenesis, they may be at timepoints that are relatively immature in other ways compared to our first trimester samples. To account for this difference, we also explored the single-cell trajectory of *Id4* from E9–E10 (our data) to E11.5–E17.5 (ref. ^47^). In the mouse, *Id4* peaks at E11.5 and is low after E13, whereas human *ID4* is sustained in its expression pattern after CS13 (Fig. 5d). By contrast, both *C1orf61* RNA and ID4 protein were detectable in macaque embryonic tissue and chimpanzee induced pluripotent stem cell-derived organoids (Supplementary Fig. 23). C1orf61 (CROC-4) has been characterized to be widely expressed during late developmental stages and in the adult brain, regulating c-FOS signaling^48^, but is otherwise uncharacterized. In contrast, *Id4* has been identified in the developing mouse cortex at later stages (E15.5). ID4-deficient mice exhibit impaired brain size and mistimed neurogenesis^49^, suggesting that earlier expression in human progenitors compared to mouse could indicate a potential mechanism by which early progenitors expand more rapidly in humans than in mice.

Despite the general conservation of cell types between the early development of the human and mouse cortex, the absence of specific human cell populations at early developmental stages in the mouse suggests that alternatives to mouse are required to fully model the earliest cell types involved in human cortical development. Cortical organoids are an attractive model because they can be generated from normal or patient-derived induced pluripotent stem cells and are amenable to genetic modulation. We previously generated an extensive catalog of single-cell sequencing data from multiple organoids derived from four pluripotent stem cell lines using three protocols analyzed from weeks 3 to 24 (ref. ^7^). Using 108,593 cells derived from weeks 3 and 5 of this dataset, we explored the fidelity of organoid cell types to early human cortex. The preservation of excitatory neuronal identity mirrored that of later developmental stages (roughly 0.5, as we recently reported). However, the remaining primary cell types had much lower fidelity in organoids (average of 0.28) (Fig. 5c). These data indicate that early cell types in organoids do not resemble their corresponding cortical progenitor counterparts, although the fidelity of cell types does improve at later stages of organoid culture. The two populations that were not well represented in mice at TS14 and 16 were identifiable in organoid cultures as evidenced by fluorescent in situ probing for *C1orf61* and immunostaining for *ID4* (Supplementary Fig. 23).

## Discussion

Here, we present a comprehensive overview of scRNA-seq from the first trimester of human development. As these samples are rarely accessible, we seek to present both the single-cell sequencing as well as the immunostaining validation provided as part of this study as a community resource. Our analysis highlights the granular gene expression programs that emerge across brain structures and across cell types in the human neocortex as development progresses, and provides a dataset by which other model organisms and in vitro cortical organoids can be compared to their primary human counterparts. The tissues used for the purpose of this study are fragile, and dissections rely on morphological hallmarks without knowledge of expression patterns before sample collection. As such, the single-cell analysis becomes even more essential as a tool to explore and validate accurate regional identities. The gene signatures that we describe and validate may offer additional markers that can be used to mark these brain regions, as well as the transition from neuroepithelia to radial glia cell identity.

Our analysis of several brain regions highlights many similarities across progenitors that were only mildly distinguished by the expression of regional-specific transcription factors. These similarities in gene programs across the developing human brain suggest that the process by which initially uniform stem cells give rise to neuronal and glia heterogeneity characteristic of the adult brain are parallel across brain structures and that insights from one region, including the degree of heterogeneity and the trajectories of differentiation, may be able to cross-inform identification of regulatory gene programs across the brain. We aspired to annotate specific gene programs that distinguish progenitor groups within the cerebral cortex. Instead, we observed a gradient of expression patterns, with clear neuroepithelial and radial glia cell populations on the ends of the spectrum, indicating that the transition from one population to another may be gradual rather than distinct. However, because our data are limited by the snapshot view of each timepoint sampled, and by imperfect representation of all possible timepoints along this continuum, this conclusion will need to be further explored through additional sampling, validation and eventual mechanistic examination.

One unexpected population that was identified in our progenitor dataset was a population that appeared mesenchymal in origin (marked by ALX1 and LUM) by both single-cell sequencing and immunostaining, that comprised a large swath of the telencephalon at the earliest timepoints that we sampled. We hypothesize that the ALX1-positive cells may resemble previously described neural crest-derived cell populations that give rise to the meninges^50^. These cells may be secreting LUM as a structural component to support the physical formation of the telencephalon before the emergence of the radial glial scaffold. In support of this hypothesis, we found that the ALX1-positive cells were in the presumptive stroma surrounding the cortex, while LUM was detected throughout the cortex. Although cortical organoids do not express ALX1 or LUM at early stages, their expression emerges after 7 weeks of development (Fig. 3b,c), further prompting questions about their function and role in cortical development.

Comparisons of primary data presented here to the single-cell sequencing of previous cortical organoid populations raises interesting differences between the systems. We were surprised to find limited cell type correspondence at the earliest stages of cortical organoid generation, and increased fidelity once the radial glia and neuronal populations emerged. This may indicate that terminal identity does not depend on a specific differentiation path. However, there were also differences in the timing and cell type composition between early human and mouse populations, further highlighting that no model system is ideal for the study of all biological questions. Moreover, populations present in primary human tissue that were not present in early organoids, including the *LUM* and *ALX1* clusters, could be identified in mice (Supplementary Figs. 18 and 19), indicating that depending on the questions being investigated, either in vivo mouse or in vitro human models, may be most appropriate for the study of neuroepithelial and early radial glia populations. Our description of early human cortical cell populations may also enable further refinement of in vitro culture systems to better reflect human neuroepithelial and radial glia populations. Together, the data we present here represent a characterization of main cell types across the first trimester of human brain development and highlight the subpopulations of progenitor cells that form the basis for creating the human cortex.

### Accession codes

The data analyzed in this study were produced through the Brain Initiative Cell Census Network (BICCN) (RRID:SCR_015820) and deposited in the Neuroscience Multi-omic (NeMO) Archive (RRID:SCR_002001), https://assets.nemoarchive.org/dat-0rsydy7.

## Methods

Additional methodological details can also be found in the Nature Research Reporting Summary included in the Supplementary Material.

### Sample processing

Acquisition of all primary human tissue samples was approved by the UCSF Human Gamete, Embryo and Stem Cell Research Committee (approval nos. 10-03379 and 10-05113). All experiments were performed in accordance with protocol guidelines. Informed consent was obtained before sample collection and use for this study. First trimester human samples were collected from elective pregnancy terminations through the Human Developmental Biology Resource, staged using crown-rump length and shipped overnight on ice in Rosewell Park Memorial Institute media or in 4% paraformaldehyde. Samples were donated and deidentified for sex. Although we can infer sex for sequenced samples, we have not accounted for it because it is an unclear measurement during the first trimester. Fixed samples were used for downstream immunostaining and imaging. Mouse samples (CD-1 IGS Mouse) were killed at E9 and E10, and staged using the somite number. Mice were housed in shared housing, five mice to a cage with a 12 light/12 dark cycle and temperatures of roughly 18–23 °C (65–75 °F) with 40–60% humidity. Equal numbers of male and female embryonic mice were used. All mouse experiments were approved by and conducted according to the UCSF Institutional Animal Care and Use Committee (protocol AN078703-03A). Half of the mouse samples were randomly assigned for fixation in 4% paraformaldehyde and the remaining live samples were used for dissociation. Live samples were subdissected into identifiable regions and dissociated using papain (Worthington, LK003150) with DNase. Samples were minced and incubated in 1 ml activated papain for 15 min at 37 °C, according to the manufacturer’s instructions. Samples were then inverted for several times and incubated for an additional 15 min. The dissociated cells were centrifuged at 300*g* for 5 min and the papain was removed. Macaque tissue sections (E64) were a gift from A. Pollen (UCSF), originally a gift from A. Tarantal (UC Davis).

### scRNA-seq

Single-cell capture was performed following the 10X v.2 Chromium manufacturer’s instructions. Each sample was its own batch. For each batch, 10,000 cells were targeted for capture and 12 cycles of amplification for each of the complementary DNA and library amplifications were performed. Libraries were sequenced according to the manufacturer’s instructions on the Illumnia NovaSeq 6000 S2 flow cell (RRID:SCR_016387).

### scRNA analysis

scRNA-seq data were aligned to the GRCh38-0.1.2 reference genome, and cells were identified using CellRanger v.2 (RRID:SCR_017344). Quality control removed cells with fewer than 500 genes per cell and cells with greater than 10% mitochondrial content. Clustering and batch effect were performed as has been previously described in these established methods^15^. Batch effects (as pertaining to day of capture) were removed by normalizing all cells in a batch to the most counts, and then multiplying by the median counts in that batch. These normalized counts were merged together across samples and log_2_ normalization was performed. Using default parameters of Seurat v.2, variable genes were identified. Batch was regressed out in the space of variable genes during scaling, again using default Seurat v.2 parameters. For three samples, there was only one individual per timepoint, which may have resulted in confounding age and batch, but because of the strong batch effects that result from 10X data, we moved ahead with the batch correction. In the space of the scaled, batch corrected variable genes, we performed principal component analysis. Significant principal components were carried forward, as identified using the calculation detailed in a previous publication. Using the RANN package, the top ten nearest neighbors were identified for each cell in the space of significant principal components. We used a custom script to calculate the Jaccard distance of these neighbors, and used igraph to perform Louvain clustering for each analysis. Clustering with batch effect correction was performed for each dissected area, and also without batch correction by each individual, both across all brain regions and within cortex only.

Cluster markers were interpreted and assigned cluster identity by using known literature cell type annotations, or by associating progenitor and neuronal genes to other identifiable details. Progenitors were distinguished as radial glia if they expressed neurogenic genes, else they were determined to be neuroepithelial (Supplementary Table 6).

Subclustering within the neurons was performed by selecting and clustering the cells from neuronal clusters and repeating the clustering procedure with batch effect correction. Progenitor cells were subclustered by removing neuronal, IPC and other (microglia, endothelial and pericyte) clusters from the data. After subclustering, neuronal populations were identified and removed iteratively until 54 subclusters were identified and did not include neuronal populations. These 54 subclusters were recombined by correlating marker genes to one another, and then performing a dendrogram cut to generate the nine clusters presented here.

Correlational analysis between mouse and human data was performed as we previously described^7^. Briefly, cluster markers were generated for each dataset individually, and gene scores integrating the specificity and fold enrichment for each marker was calculated. A matrix for every marker gene and gene score was generated across all clusters and used for correlations. This was performed in the space of all mouse forebrain clusters as compared to primary human progenitor clusters (Fig. 5), and in the space of all organoid clusters compared to the full set of primary human clusters in our dataset (Supplementary Fig. 23).

### RNA velocity

Velocity estimates were calculated using the veloctyo.py v0.17 and scVelo (RRID:SCR_018168) algorithms. Reads that passed quality control after clustering were used as input for the velocyto command line implementation. The human expressed repeat annotation file was retrieved from the UCSC genome browser (RRID:SCR_005780). The genome annotation file used was provided via CellRanger. The output loom file was used as input to estimate velocity through scVelo. For each individual analysis, cells were filtered based on the following parameters: minimum total counts ≥200, minimum spliced counts ≥20 and minimum unspliced counts ≥10. For the combined cortical analysis, the processed loom files for each individual analysis were combined to generate a new unique molecular identifier count matrix of 15,473 genes across 53,096 cells, for which the velocity embedding was estimated using the stochastic model. For the combined progenitor analysis, cells that were identified as progenitors were used to create the loom file. The loom files for each of the individuals were combined for a total count matrix of 14,207 genes across 30,562 cells for the velocity embedding using the same criteria. Each embedding was visualized using uniform manifold approximation and projection (UMAP) of dimension reduction.

### Immunostaining

Primary human and mouse samples and organoids were collected, fixed in 4% paraformaldehyde, washed with 1× PBS and immersed in 30% sucrose in 1× PBS until saturated. Samples were embedded in cryomolds using 50% optimal temperature cutting (OCT) compound (Tissue-Tek catalog no. 4583) and 50% of 30% sucrose in 1× PBS and frozen at −80 °C. All samples were sectioned at 16 μm onto SuperFrost Plus microscope slides. Citrate antigen retrieval (Vector Laboratories, catalog no. H-3300) was performed for 20 min at roughly 95–100 °C. Slides were then washed with 1× PBS and blocked using 5% donkey serum, 2% gelatin, 0.1% Triton in 1× PBS for 90 min at room temperature. Primary antibody incubation occurred in blocking buffer overnight at 4 °C, and washed five times using 0.3% Triton + 10 mM glycine. Secondary antibody incubation occurred in blocking buffer for 2–3 h at room temperature. Primary antibodies used were TRKC (1:200, R and D Systems catalog no. AF373, RRID:AB_355332); ALX1 (1:500, Santa Cruz Biotechnology catalog no. sc-130416, RRID:AB_2226324); ID4 (1:200, Santa Cruz Biotechnology catalog no. sc-365656, RRID:AB_10859382); N-CADHERIN (1:300, Abcam catalog no. ab18203, RRID:AB_444317); DLK1 (1:200, Abcam catalog no. ab119930, RRID:AB_10902607); DLK1 (1:100, Abcam catalog no. ab21682, RRID:AB_731965); CROC-4 (1:100, Aviva Systems catalog no. ARP34802_P050, RRID:AB_2827813); LUM (1:50, Thermo Fisher Scientific catalog no. MA5-29402, RRID:AB_2785270); ZO-1 (1:100, Thermo Fisher Scientific catalog no. 61-7300, RRID:AB_2533938); SOX2 (1:100, R and D Systems catalog no. AF2018, RRID:AB_355110); SOX2 (1:250, Santa Cruz Biotechnology catalog no. sc-365823, RRID:AB_10842165); CTIP2 (1:500, Abcam catalog no. ab18465, RRID:AB_2064130); KI67 (1:200, Thermo Fisher Scientific catalog no. 14-5698, RRID: AB_10854564); HOPX (1:250, Santa Cruz Biotechnology catalog no. sc-398703, RRID:AB_2687966); HOPX (1:200, Proteintech catalog no. 11419-1-AP, RRID:AB_10693525); TBR2 (1:250, Abcam catalog no. ab23345, RRID:AB_778267); TBR2 (1:250, R and D Systems catalog no. AF6166, RRID:AB_10569705); NESTIN (1:200, Millipore catalog no. MAB5326, RRID:AB_2251134), DCX (1:500, Aves Laboratories catalog no. DCX, RRID:AB_2313540); NEUN (1:250, Millipore catalog no. ABN91, RRID:AB_11205760); PAX6 (1:200, BioLegend catalog no. 901301, RRID:AB_2565003); FOXG1 (1:1000, Abcam catalog no. ab18259, RRID:AB_732415); SATB2 (1:250, Abcam catalog no. Ab51502, RRID:AB_882455); LHX5 (1:100, R and D Systems catalog no. AF6290, RRID:AB_10973257); REELIN (1:100, MBL catalog no. D223-3, RRID:AB_843523); Phospho-β-CATENIN (1:100, Cell Signaling Technology catalog no. 9561, RRID:AB_331729); Phospho-S6 (1:100, Cell Signaling Technology catalog no. 2211S, RRID:AB_331679) and NICD/NOTCH1 (1:100, Millipore catalog no. 07-1232, RRID:AB_1977387). All secondary antibodies were AlexaFluor used at a dilution of 1:1,000. Secondary antibodies used were donkey antimouse 488 (Thermo Fisher Scientific catalog no. A32766, RRID:AB_2762823); donkey antirabbit 488 (Thermo Fisher Scientific catalog no. A32790, RRID:AB_2762833); donkey antichicken 488 (Jackson ImmunoResearch Laboratories catalog no. 703-545-155, RRID:AB_2340375); donkey antichicken 594 (Jackson ImmunoResearch Laboratories catalog no. 703-585-155, RRID:AB_2340377); donkey antimouse 546 (Thermo Fisher Scientific catalog no. A10036, RRID:AB_2534012); donkey antimouse 594 (Thermo Fisher Scientific catalog no. A-21203, RRID:AB_141633); donkey antimouse 647 (Thermo Fisher Scientific catalog no. A32787, RRID:AB_2762830); donkey antimouse 680 (Thermo Fisher Scientific catalog no. A32788, RRID:AB_2762831); donkey antirabbit 546 (Thermo Fisher Scientific catalog no. A10040, RRID:AB_2534016); donkey antirabbit 594 (Thermo Fisher Scientific catalog no. A-21207, RRID:AB_141637); donkey antirabbit 647 (Thermo Fisher Scientific catalog no. A32795, RRID:AB_2762835); donkey antirat 594 (Thermo Fisher Scientific catalog no. A-21209, RRID:AB_2535795); donkey antirat 488 (Thermo Fisher Scientific catalog no. A-21208, RRID:AB_2535794); donkey antigoat 546 (Thermo Fisher Scientific catalog no. A-11056, RRID:AB_2534103); donkey antigoat 594 (Thermo Fisher Scientific catalog no. A-11058, RRID:AB_2534105); donkey antigoat 647 (Thermo Fisher Scientific catalog no. A32849, RRID:AB_2762840); donkey antisheep 546 (Thermo Fisher Scientific catalog no. A-21098, RRID:AB_2535752); donkey antisheep 594 (Thermo Fisher Scientific catalog no. A-11016, RRID:AB_2534083); donkey antisheep 647 (Thermo Fisher Scientific catalog no. A-21448, RRID:AB_2535865) and donkey antiguinea pig 647 (Jackson ImmunoResearch Laboratories catalog no. 706-605-148, RRID:AB_2340476).

### In situ hybridization

Primary fixed samples were treated using the protocol for RNAScope Multiplex Fluorescence Assay v.2 (Advanced Cell Diagnostics catalog no. 323100) for *C1orf61* amplification, targeting nucleotides 60–897 of NM_006365.3 (Advanced Cell Diagnostics Probe Design no. NPR-0003991); MEF2C amplification, targeting nucleotides 1058–2575 of NM_002397.4 (Advanced Cell Diagnostics catalog no. 452881) and the protocol for BaseScope v.2 Assay (Advanced Cell Diagnostics catalog no. 323900) for chromogenic LHX5-AS1 amplification, targeting nucleotides 69–293 of NR_126425.1 (Advanced Cell Diagnostics Probe Design no. NPR-0003991).

### Imaging and image processing

Images were collected on the Leica SP8 (RRID:SCR_018169) inverted confocal microscope using a ×40 oil-immersion objective. Because of the scarcity of first trimester primary tissue, only one sample per panel was imaged. For each imaging panel, the parameters (including the gain, offset, pinhole and laser power) for image acquisition was left constant for all samples. Images were later processed using FIJI Image J (RRID:SCR_003070).

### Statistical tests

No statistical methods were used to predetermine sample sizes. No randomization was used in this study. Distributions of the data were not tested. Data collection and analysis were not performed blind to the conditions of the experiments. The Wilcoxon rank sum test was used to calculate cluster markers within Seurat for a variety of analyses. A one-sided *t*-test was used in Supplementary Figs. 1e and 15c. A loess regression was used to estimate smoothed gene expression in Supplementary Fig. 11 and Fig. 4.

### Reporting Summary

Further information on research design is available in the Nature Research Reporting Summary linked to this article.

## Online content

Any methods, additional references, Nature Research reporting summaries, source data, extended data, supplementary information, acknowledgements, peer review information; details of author contributions and competing interests; and statements of data and code availability are available at 10.1038/s41593-020-00794-1.

## Supplementary information

Supplementary Information Supplementary Figs. 1–26.

Reporting Summary

Supplementary Tables Supplementary Tables 1–12.

## Data Availability

The data that support the findings of this study are available from the corresponding author upon request. Raw single-cell sequencing data are available from the NeMO Repository at https://assets.nemoarchive.org/dat-0rsydy7. Processed single-cell sequencing data and full tilescan images are available for exploration and for download at our cell browser: https://cells-test.gi.ucsc.edu/?ds=early-brain.

## Code availability

No custom code was used in this study. Open source algorithms were used as detailed in single-cell analysis methods. However, any details on how these algorithms were used are available from the corresponding author upon request.
