# Supplementary material for: Single-cell atlas of early human brain development highlights heterogeneity of human neuroepithelial cells and early radial glia
Source: Nat Neurosci. 2021 Mar 15;24(4):584–94. doi: 10.1038/s41593-020-00794-1 (PMC8012207; doi:10.1038/s41593-020-00794-1)
Supplement: Supplementary file 2 — Reporting Summary [file 41593_2020_794_MOESM2_ESM.pdf]

nature research

Corresponding author(s): Arnold Kriegstein and Aparna Bhaduri

Last updated by author(s): Nov 16, 2020

Reporting Summary

Nature Research wishes to improve the reproducibility of the work that we publish. This form provides structure for consistency and transparency in reporting. For further information on Nature Research policies, see Authors & Referees and the Editorial Policy Checklist.

Statistics

For all statistical analyses, confirm that the following items are present in the figure legend, table legend, main text, or Methods section.

n/a	Confirmed	
☐	☒	The exact sample size (n) for each experimental group/condition, given as a discrete number and unit of measurement
☐	☒	A statement on whether measurements were taken from distinct samples or whether the same sample was measured repeatedly
☐	☒	The statistical test(s) used AND whether they are one- or two-sided<br>*Only common tests should be described solely by name; describe more complex techniques in the Methods section.*
☐	☒	A description of all covariates tested
☐	☒	A description of any assumptions or corrections, such as tests of normality and adjustment for multiple comparisons
☐	☒	A full description of the statistical parameters including central tendency (e.g. means) or other basic estimates (e.g. regression coefficient) AND variation (e.g. standard deviation) or associated estimates of uncertainty (e.g. confidence intervals)
☐	☒	For null hypothesis testing, the test statistic (e.g. F, t, r) with confidence intervals, effect sizes, degrees of freedom and P value noted<br>*Give P values as exact values whenever suitable.*
☒	☐	For Bayesian analysis, information on the choice of priors and Markov chain Monte Carlo settings
☒	☐	For hierarchical and complex designs, identification of the appropriate level for tests and full reporting of outcomes
☒	☐	Estimates of effect sizes (e.g. Cohen's d, Pearson's r), indicating how they were calculated

Our web collection on statistics for biologists contains articles on many of the points above.

Software and code

Policy information about availability of computer code

Data collection	No specialized software or code was used for data collection
Data analysis	Data analysis was performed with Cellranger v2, Seurat v2, R packages, scVelo and other open source packages as indicated in the Methods

For manuscripts utilizing custom algorithms or software that are central to the research but not yet described in published literature, software must be made available to editors/reviewers. We strongly encourage code deposition in a community repository (e.g. GitHub). See the Nature Research guidelines for submitting code & software for further information.

Data

Policy information about availability of data

All manuscripts must include a data availability statement. This statement should provide the following information, where applicable:

- Accession codes, unique identifiers, or web links for publicly available datasets
- A list of figures that have associated raw data
- A description of any restrictions on data availability

The raw data for the provided figures is available via the UCSC Image Browser [https://early-brain.cells.ucsc.edu, Images tab], at this link the RNA-seq processed files are also available. The raw data is also available through dbGAP and the NeMO repository: https://assets.nemoarchive.org/dat-0rsydy7.

Other data used include the GRCh38-0.1.2 reference genome, downloaded with cellranger v2.

Field-specific reporting

Please select the one below that is the best fit for your research. If you are not sure, read the appropriate sections before making your selection.

☒ Life sciences ☐ Behavioural & social sciences ☐ Ecological, evolutionary & environmental sciences

For a reference copy of the document with all sections, see nature.com/documents/nr-reporting-summary-flat.pdf

Life sciences study design

All studies must disclose on these points even when the disclosure is negative.

Sample size	Because of the scarcity of first trimester human samples, we used one sample per age for each panel of figures. For the organoid and mouse samples, the data imaged represents a sample of 1. The single-cell data for the mouse samples represents a sample size of 3 individuals. Sample sizes were chosen based upon the ability to get representative data with the number of single-cells for the comparison. As such, we had 289,000 cells from the human and 16053 cells from mouse which is sufficient to identify cell types based upon numerous studies in the field.
Data exclusions	In all the single-cell analyses, we excluded all cells that had fewer than 500 genes per cell and had greater than 10% mitochondrial content. However, these data are available from the raw data. These exclusions were pre-established and were necessary to eliminate droplets that might not contain actual cells or that contain dead cells from downstream analysis.
Replication	All imaged primary human data had one replicate due to the scarcity of the tissue. For the single-cell data of the human tissue, there were two Carnegie Stage 14 , Carnegie Stage 15 and Carnegie Stage 22 replicates each. There was one replicate for the remaining ages due to limited tissue availability. Each imaged mouse and organoid sample represents one replicate. All attempts for single-cell collection of data were successful.
Randomization	In each immunostaining panel, the same tissue sample from one individual was sectioned and slices were evenly distributed across all imaging panel conditions. No randomization was performed across other analysis, and this was not relevant because the data was processed with pre-determined conditions and compared between methods (different individuals, immunostaining, etc).
Blinding	Blinding was not performed, and it was not meaningful because only one sample was collected at one point. During analysis, blinding was not meaningful because all samples were treated equally by pre-determined analyses and thresholds. However, we used clearly defined imaging and image processing criteria (described in our methods) for all our analyses and analyzed all included samples using the same rigorous criteria in order to avoid bias.

Reporting for specific materials, systems and methods

We require information from authors about some types of materials, experimental systems and methods used in many studies. Here, indicate whether each material, system or method listed is relevant to your study. If you are not sure if a list item applies to your research, read the appropriate section before selecting a response.

Materials & experimental systems

n/a	Involved in the study	
☐	☒	Antibodies
☐	☒	Eukaryotic cell lines
☒	☐	Palaeontology
☐	☒	Animals and other organisms
☐	☒	Human research participants
☒	☐	Clinical data

Methods

n/a	Involved in the study	
☒	☐	ChIP-seq
☒	☐	Flow cytometry
☒	☐	MRI-based neuroimaging

Antibodies

Antibodies used	All secondary antibodies were AlexaFluor used at a dilution 1:1000. Secondary antibodies: Donkey anti-Mouse 488 (Thermo Fisher Scientific Cat# A32766, RRID:AB_2762823); Donkey anti-Rabbit 488 (Thermo Fisher Scientific Cat# A32790, RRID:AB_2762833); Donkey anti-Chicken 488 (Jackson ImmunoResearch Labs Cat# 703-545-155, RRID:AB_2340375); Donkey anti-chicken 594 (Jackson ImmunoResearch Labs Cat# 703-585-155, RRID:AB_2340377); Donkey anti-Mouse 546 (Thermo Fisher Scientific Cat# A10036, RRID:AB_2534012); Donkey anti-Mouse 594 (Thermo Fisher Scientific Cat# A-21203, RRID:AB_141633); Donkey anti-Mouse 647 (Thermo Fisher Scientific Cat# A32787, RRID:AB_2762830); Donkey anti-Mouse 680 (Thermo Fisher Scientific Cat# A32788, RRID:AB_2762831); Donkey anti-Rabbit 546 (Thermo Fisher Scientific Cat# A10040, RRID:AB_2534016); Donkey anti-Rabbit 594 (Thermo Fisher Scientific Cat# A-21207, RRID:AB_141637); Donkey anti-Rabbit 647 (Thermo Fisher Scientific Cat# A32795, RRID:AB_2762835); Donkey anti-Rat 594 (Thermo Fisher Scientific Cat# A-21209, RRID:AB_2535795); Donkey anti-Rat 488 (Thermo Fisher Scientific Cat# A-21208, RRID:AB_2535794); Donkey anti-Goat 546 (Thermo Fisher Scientific Cat# A-11056, RRID:AB_2534103); Donkey anti-Goat 594 (Thermo Fisher Scientific Cat# A-11058, RRID:AB_2534105); Donkey anti-Goat 647 (Thermo Fisher Scientific Cat# A32849, RRID:AB_2762840); Donkey anti-Sheep 546 (Thermo Fisher Scientific Cat#

A-21098, RRID:AB_2535752); Donkey anti-Sheep 594 (Thermo Fisher Scientific Cat# A-11016, RRID:AB_2534083); Donkey anti-Sheep 647 (Thermo Fisher Scientific Cat# A-21448, RRID:AB_2535865); Donkey anti-Guinea Pig 647 (Jackson ImmunoResearch Labs Cat# 706-605-148, RRID:AB_2340476).

TRKC (1:200, R and D Systems Cat# AF373, RRID:AB_355332); ALX1 (1:500, Santa Cruz Biotechnology Cat# sc-130416, RRID:AB_2226324); ID4 (1:200, Santa Cruz Biotechnology Cat# sc-365656, RRID:AB_10859382); N-CADHERIN (1:300, Abcam Cat# ab18203, RRID:AB_444317); DLK1 (1:200, Abcam Cat# ab119930, RRID:AB_10902607); DLK1 (1:100, Abcam Cat# ab21682, RRID:AB_731965); CROC-4 (1:100, Aviva Systems Cat# ARP34802_P050, RRID:AB_2827813); LUM (1:50, Thermo Fisher Scientific Cat# MA5-29402, RRID:AB_2785270); ZO-1 (1:100, Thermo Fisher Scientific Cat# 61-7300, RRID:AB_2533938); SOX2 (1:100, R and D Systems Cat# AF2018, RRID:AB_355110); SOX2 (1:250, Santa Cruz Biotechnology Cat# sc-365823, RRID:AB_10842165); CTIP2 (1:500, Abcam Cat# ab18465, RRID:AB_2064130); KI67 (1:200, Thermo Fisher Scientific Cat# 14-5698, RRID: AB_10854564); HOPX (1:250, Santa Cruz Biotechnology Cat# sc-398703, RRID:AB_2687966); HOPX (1:200, Proteintech Cat# 11419-1-AP, RRID:AB_10693525); TBR2 (1:250, Abcam Cat# ab23345, RRID:AB_778267); TBR2 (1:250, R and D Systems Cat# AF6166, RRID:AB_10569705); NESTIN (1:200, Millipore Cat# MAB5326, RRID:AB_2251134), DCX (1:500, Aves Labs Cat# DCX, RRID:AB_2313540); NEUN (1:250, Millipore Cat# ABN91, RRID:AB_11205760); PAX6 (1:200, BioLegend Cat# 901301, RRID:AB_2565003); FOXG1 (1:1000, Abcam Cat# ab18259, RRID:AB_732415); SATB2 (1:250, Abcam Cat# Ab51502, RRID:AB_882455); LHX5 (1:100, R and D Systems Cat# AF6290, RRID:AB_10973257); REELIN (1:100, MBL Cat# D223-3, RRID:AB_843523); Phospho-B-CATENIN (1:100, Cell Signaling Technology Cat# 9561, RRID:AB_331729); Phospho-S6 (1:100, Cell Signaling Technology Cat# 2211S, RRID:AB_331679); NICD/NOTCH1 (1:100, Millipore Cat# 07-1232, RRID:AB_1977387).

Validation

Validation:
TRKC R&D Systems, AF373: Western Blot validation at 164 kDa using the 12-230 kDA separation system under reducing conditions https://www.rndsystems.com/products/human-trkc-antibody_af373

ALX1 Santa Cruz, sc-130416:raised agaisnt recombinant ALX1 of human origin. Western Blot analysis shows validation at the ~44kDa. https://www.scbt.com/p/alx1-antibody-96k

ID4 Santa Cruz, sc-365656: raised against amino acids 1-70 mapping the N-terminus of Id4 of human origin. Western blot validaiton at ~20kDa. https://www.scbt.com/p/id4-antibody-b-5.

N-CADHERIN Abcam, ab18203: Synthetic peptide corresponding to Human N Cadherin aa 800-900 (internal sequence) conjugated to keyhole limpet haemocyanin. Western blot validation at 125 kDA. https://www.abcam.com/n-cadherin-antibody-ab18203.html

DLK1 Abcam, ab119930: Recombinant fragment corresponding to Human DLK-1 aa 174-349. Western blot validation at 41 kDa. https://www.abcam.com/dlk-1-antibody-3a10-ab119930.html

DLK1 Abcam, ab21682: Synthetic peptide corresponding to Human DLK-1 aa 350 to the C-terminus (C terminal) conjugated to keyhole limpet haemocyanin. Western Blot validation at 45 and 48 kDa. https://www.abcam.com/dlk-1-antibody-ab21682.html

CROC-4 Aviva Systems, ARP34802_P050: a synthetic peptide directed towards the N-terminal region of Human CROC4. Western Blot validation at ~35 and 22 kDA. https://www.avivasysbio.com/c1orf61-antibody-n-terminal-region-arp34802-p050.html

LUM Thermo Fisher, MA5-29402: recombinant protein targeting the recombinant human lumican protein. https://www.thermofisher.com/antibody/product/LUM-Antibody-clone-77-Recombinant-Monoclonal/MA5-29402

ZO-1 Thermo Fisher, 61-7300: A 69 kD fusion protein(1) corresponding to amino acids 463-1109 of human ZO-1 cDNA.(2) This sequence lies N-terminal to the 80 amino acid region (the alpha-motif) present in the a+-isoform but absent in the a- isoform due to alternative splicing. https://www.thermofisher.com/antibody/product/ZO-1-Antibody-Polyclonal/61-7300

SOX2 R&D Systems, AF2018: Polyclonal antibody raised against recombinant human SOX2. Western Blot validation at 36 kDa. https://www.rndsystems.com/products/human-mouse-rat-sox2-antibody_af2018

SOX2 Santa Cruz, sc-365823: specific for an epitope mapping between amino acids 170-201 within an internal region of Sox-2 of human origin. Western Blot validation at ~36 kDa. https://www.scbt.com/p/sox-2-antibody-e-4

CTIP2 Abcam, ab18465: Detects 2 bands representing Ctip2 at about 120kD (between aa 1-150 kDa). Western blot validation at ~128 and 129 kDa. https://www.abcam.com/ctip2-antibody-25b6-chip-grade-ab18465.html

KI67 Thermo Fisher, 14-5698: monoclonal antibody recognizes mouse and rat Ki-67, a 300 kDa nuclear protein. This Antibody was verified by Cell treatment to ensure that the antibody binds to the antigen stated. https://www.thermofisher.com/antibody/product/Ki-67-Antibody-clone-SolA15-Monoclonal/14-5698-82

HOPX Santa Cruz, sc-398703: raised against amino acids 1-73 representing full length Hop of human origin. Western Blot validation at ~15 kDa. ttps://www.scbt.com/p/hop-antibody-e-1

HOPX Proteintech, 11419-1-AP. HopX fusion protein. Western Blot validation at ~ 10kDa. https://www.ptglab.com/Products/HOPX-Antibody-11419-1-AP.htm

TBR2 Abcam, ab23345: Synthetic peptide corresponding to Mouse TBR2/ Eomes aa 650 to the C-terminus (C terminal) conjugated to keyhole limpet haemocyanin. Western blot validation at 85 kDa. https://www.abcam.com/tbr2-eomes-antibody-chip-grade-ab23345.html

TBR2 R&D Systems, AF6166: E. coli-derived recombinant human EOMES. Western blot validation at ~95 and 96 kDa. https://www.rndsystems.com/products/human-eomes-antibody_af6166

NESTIN Millipore Sigma, MAB5326: clone 10C2 for detection Nestin fusion protein. Western blot validation at 220 -240 kDa. https://www.sigmaaldrich.com/catalog/product/mm/mab5326?lang=en&region=US

DCX Aves Labs, DCX: Two antipeptide antibodies were generated in chickens against sequences shared between the mouse (AAT58219.1), rat (NP_445831.3) and human (CAA06617.1) gene products. https://www.aveslabs.com/products/doublecortin

NEUN, Millipore Sigma, ABN91: GST-tagged recombinant protein corresponding to the N-terminus of mouse NeuN. Western Blot validationa t ~45kDa. https://www.sigmaaldrich.com/catalog/product/mm/abn91?lang=en&region=US

PAX6 Biolegend, 901301: antibody was generated against the peptide (QVPGSEPDMSQYWPRLQ) derived from the C-terminus of the mouse Pax-6 protein. Western Blot validation at 46.6 and 48.2 kDa.https://www.biolegend.com/en-us/products/purified-anti-pax-6-antibody-11511

FOXG1 Abcam, ab18259: Synthetic peptide corresponding to Human FOXG1 aa 400 to the C-terminus (C terminal) conjugated to keyhole limpet haemocyanin. Western Blot validation at 50 kDa. https://www.abcam.com/foxg1-antibody-chip-grade-ab18259.html

SATB2 Abcam, ab51502: Recombinant fragment corresponding to the C-terminal of Human SATB2. Western blot validation at 82kDa. https://www.abcam.com/satb2-antibody-satba4b10-c-terminal-ab51502.html

LHX5 R and D Systems, AF6290: Detects recombinant human LHX5 at 55-60 kDa in Western Blot. https://www.rndsystems.com/products/human-mouse-rat-lhx5-antibody_af6290

REELIN MBL, D223-3: reacts with mouse Reelin. The CR-50 epitope is located between mouse Reelin amino acid 230 to 346 4. https://www.mblintl.com/products/d223-3/

Phospho-B-Catenin Cell Signaling, 9561: detects endogenous levels of β-catenin only when phosphorylated at serines 33, 37 or threonine 41. Polyclonal antibodies are produced by immunizing animals with a synthetic phosphopeptide corresponding to residues surrounding Ser33, Ser37 and Thr41 of human B-catenin. https://www.cellsignal.com/products/primary-antibodies/phospho-b-catenin-ser33-37-thr41-antibody/

Phospho-S6 Cell Signaling, 2211S: detects endogenous levels of ribosomal protein S6 only when phosphorylated at serine 235 and 236. Polyclonal antibodies are produced by immunizing animals with a synthetic phosphopeptide corresponding to residues surrounding Ser235 and Ser236 of human ribosomal protein S6. https://www.cellsignal.com/products/primary-antibodies/phospho-s6-ribosomal-protein-ser235-236-antibody/

NICD Millipore, 07-1232: Notch 1, cleaved N terminal. Only the cleaved intracellular (activated) form is detected. Synthetic peptide from the N-terminal sequence of the cleaved N intracellular domain (NICD) human Notch 1. Western blot detection at 80 kDa. https://www.sigmaaldrich.com/catalog/product/mm/071232?lang=en&region=US

Eukaryotic cell lines

Policy information about cell lines

Cell line source(s)	H1 (WA01) embryonic stem cell line (source: WiCell)<br>1323-4 induced pluripotent stem cell line (source: Bruce Conklin, Gladstone Institute)<br>H28126 induced pluripotent stem cell line (source: Yoav Gilad, University of Chicago)<br>4955 induced pluripotent stem cell line (source: Yoav Gilad, University of Chicago)
Authentication	Each stem cell line was karyotyped and validated for pluripotency, prior to receipt. Every 10 passages, stem cells are tested for karyotypic abnormalities and validated for pluripotency markers Sox2, Nanog, and Oct4.
Mycoplasma contamination	All cell lines tested negative for mycoplasma.
Commonly misidentified lines (See ICLAC register)	No commonly misidentified lines were used.

Animals and other organisms

Policy information about studies involving animals; ARRIVE guidelines recommended for reporting animal research

Laboratory animals	CD-1® IGS Mouse, sacrificed at E9 and E10 with equal numbers of male and female embryonic mice used. Housing conditions are described in the Methods.
Wild animals	No wild animals were used in this study.
Field-collected samples	No field-collected samples were used in this study.
Ethics oversight	All mouse experiments were approved by and conducted according to the UCSF Institutional Animal Care and Use Committee (protocol AN078703-03A).

Note that full information on the approval of the study protocol must also be provided in the manuscript.

Human research participants

Policy information about studies involving human research participants

Population characteristics	Because of the sensitivity of the samples, no population characteristics are known or recorded.
Recruitment	No recruitment criteria other than consent were required.
Ethics oversight	Acquisition of all primary human tissue samples was approved by the UCSF Human Gamete, Embryo and Stem Cell Research Committee (GESCR, approval 10-03379 and 10-05113). All experiments were performed in accordance with protocol guidelines. Informed consent was obtained before sample collection and use for this study.

Note that full information on the approval of the study protocol must also be provided in the manuscript.
